# Freestanding bilayer microscope for single-molecule imaging of membrane proteins

**DOI:** 10.1126/sciadv.ado4722

**Published:** 2024-06-21

**Authors:** Gonzalo Pérez-Mitta, Yeliz Sezgin, Weiwei Wang, Roderick MacKinnon

**Affiliations:** Laboratory of Molecular Neurobiology and Biophysics, Howard Hughes Medical Institute, The Rockefeller University, New York, NY, USA.

## Abstract

Integral membrane proteins (IMPs) constitute a large fraction of organismal proteomes, playing fundamental roles in physiology and disease. Despite their importance, the mechanisms underlying dynamic features of IMPs, such as anomalous diffusion, protein-protein interactions, and protein clustering, remain largely unknown due to the high complexity of cell membrane environments. Available methods for in vitro studies are insufficient to study IMP dynamics systematically. This publication introduces the freestanding bilayer microscope (FBM), which combines the advantages of freestanding bilayers with single-particle tracking. The FBM, based on planar lipid bilayers, enables the study of IMP dynamics with single-molecule resolution and unconstrained diffusion. This paper benchmarks the FBM against total internal reflection fluorescence imaging on supported bilayers and is used here to estimate ion channel open probability and to examine the diffusion behavior of an ion channel in phase-separated bilayers. The FBM emerges as a powerful tool to examine membrane protein/lipid organization and dynamics to understand cell membrane processes.

## Teaser

The freestanding bilayer microscope is a single-molecule microscope to study cell membrane processes in vitro.

## INTRODUCTION

Integral membrane proteins (IMPs) encode 20 to 30% of the proteomes of organisms across all domains of life (*1*, *2*). Receptors, transporters, and channels compose most IMPs in humans and are about 60% of all therapeutic drug targets, exemplifying the importance of these proteins in physiology and disease (*3*). However, many dynamic features of IMPs remain enigmatic and controversial. For example, IMPs in the plasma membrane switch stochastically between diverse motions when observed under a microscope; the same protein can transition from a random walk to a linear motion or a complete stop (*4*, *5*). Simultaneously, IMPs can interact with themselves and other proteins and adopt nonhomogeneous distributions across membranes (*6*, *7*). The origin of such features needs to be addressed to develop good dynamical models of cell membranes.

Explaining these phenomena requires curtailing some of the intrinsic complexities of the cellular environment. This can be achieved with experimental setups that allow the study of IMPs in membranes of controlled composition with single-particle tracking (SPT) (*8*, *9*). SPT permits the study of the diverse aspects of IMP dynamics, such as anomalous diffusion, dynamic heterogeneity, and protein-protein interactions, by obtaining the full information contained in particle positions over the course of a time-lapse experiment (*10*–*12*).

Numerous methods exist to study membrane proteins in isolation from their cellular environments, including detergent micelles, nanodiscs, vesicles, and planar bilayers. Of these, the ones suitable for studying dynamics, such as diffusion or intermolecular reactions, where large patches of flat membranes are required, can be classified into supported bilayer (SB) and freestanding bilayer (FB) techniques. SB methods require forming a membrane on top of a solid (or gel) substrate (*13*–*16*). Despite being easier to implement, these methods are inadequate for the study of IMPs because proteins are strongly affected by the presence of the substrate (*17*, *18*). FB methods, where the membrane is suspended in solution, avoid immobilizing proteins, but current implementations of FBs have relied mostly on confocal optics that are not compatible with SPT (*19*, *20*).

Here, we present the Freestanding Bilayer microscope (FBM)—a technique that combines the advantages of FBs with SPT. We based our instrument on planar bilayers (PBs) for several reasons (*21*). PBs have been shown to preserve the function of many ion channels of diverse types and organisms (*22*–*25*). Also, PBs have diameters of hundreds of micrometers, which makes them ideal for imaging, and both sides of the membrane are accessible, allowing for great versatility in experimental design. Scanning a focused laser to illuminate the bilayer enabled us to observe fast diffusing IMPs at single-molecule resolution. We showed that, in our system, IMPs diffuse freely as opposed to SBs. We then showed how the FBM can be applied to answer diverse biological questions with two examples: the determination of the open probability of an ion channel by performing simultaneous imaging and electrical recordings and the study of the dynamic organization of proteins and lipids by tracking diffusing IMPs in phase-separated lipid bilayers.

## RESULTS

### Freestanding bilayer microscope design

**Fig. 1.**
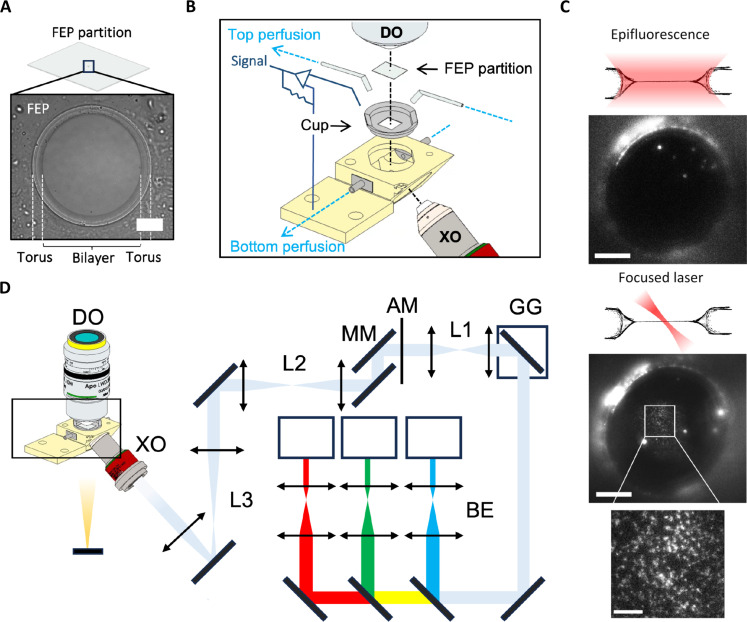
Design and characterization of the FBM. (**A**) Image of a representative planar bilayer observed under transillumination. The boundaries between the bilayer, the Plateau-Gibbs border (torus), and the fluorinated ethylene propylene (FEP) partition can be seen. The bilayer composition is POPE:POPG (3:1 by weight). Scale bar, 50 μm. (**B**) Amplified view of a technical illustration of the imaging chamber. Planar bilayers are formed across perforated FEP copolymer films (FEP partitions) mounted on 3D-printed sample holders (cups). Cups attach to the experimental chamber, shown in yellow, delimiting the top and bottom sides. The chamber provides access to the excitation objective (XO) at 39° with respect to the optical plane of the bilayer, a transillumination source, and perfusion adaptors that can be connected to a perfusion system (bottom perfusion) or to a manometer to regulate the curvature of the bilayer. A different set of perfusion adaptors (top perfusion) are added to the top chamber along with the detection objective (DO). Ag/AgCl electrodes are added to the top and bottom sides for electrophysiological recordings. (**C**) Fluorescent images taken with the FBM under epifluorescence (top) and focused-laser (FL) illumination (bottom) of fluorescently labeled hTRAAK. Comparison between both illumination sources shows that resolved diffraction-limited particles can only be observed using FL illumination. The inset shows a magnified view of the FL illumination. Scale bars, (epi) 50 μm, (FL) 50 μm, and (inset) 10 μm. hTRAAK was labeled with LD655-NHS. (**D**) Scheme of the FBM depicting the relation between the main components of the system. Lasers (473, 561, and 660 nm) are fed to three beam expanders (BE) and optically conjugated to a galvo-galvo (GG) scanner, an apodization mask (AM), and to the back focal plane of an XO through three optical relays (L1 to L3). Photons emitted from the sample are collected by a DO.

We designed the FBM to observe membrane proteins in PBs. PBs are formed by adding a small amount of lipid solution in an organic solvent (decane), across a hole in a plastic film (partition) submerged in an aqueous solution. We have found that decane leads to the formation of stable bilayers and to the functional reconstitution of many different ion channels and that is why we use it as our solvent of choice. We expect no major differences if other *n*-alkanes are used instead. The interfacial forces between the organic solvent, water, and the plastic material lead to the formation of a lipid bilayer at the center of the hole held in place by a Plateau-Gibbs border of lipid and solvent (torus) (Fig. 1A). For the partitions, we used fluorinated ethylene propylene (FEP) because it was the only material that fulfilled the conditions of low autofluorescence, good bilayer-forming properties, low refractive index, and transparency required for our experiments. Partitions were cut and perforated at the center, creating ~200-μm-diameter holes to support the bilayers, and were then mounted on a sample holder (cup) using vacuum grease to prevent leaks between the bottom and top chambers (Fig. 1B). The cups attach to the experimental chamber, which is itself mounted on a micromanipulator and was designed to contain the tip of the excitation objective (XO) as well as ports for the perfusion of solution (bottom perfusion) (Fig. 1B). The bottom perfusion ports are also connected to a manometer used to regulate the curvature of the bilayer (fig. S1). Another set of perfusion channels was added to the cup to perfuse the top chamber (Fig. 1B). To observe the bilayer under transmitted light, a glass window was included at the bottom of the chamber.

Ag/AgCl electrodes were added to the top and bottom chambers to record currents across the bilayer to monitor the formation of the bilayer (sharp increase in capacitance) and to record the activity of ion channels in the bilayer (Fig. 1B).

Since the PBs offer a natural optical sectioning of the emission of fluorescent proteins within it, we expected that an epifluorescence source would suffice to detect single molecules. However, the emission from the torus surrounding the bilayer caused a high background that prevented particle detection (Fig. 1C, fig. S2A, and movie S1). Inspired by the selective plane illumination microscopes, we designed a scanned focused laser illumination that achieves precise illumination of fluorescent probes at the bilayer (Fig. 1, C and D, and fig. S2B) (*26*–*28*). Focused illumination allowed us to observe single particles on the bilayer at typical frame rates of 50 Hz (Fig. 1C and movie S2). To achieve this, the lasers were first expanded and combined and then focused through a water immersion and long working distance (WD) objective at the bilayer (XO), positioned at an angle of 39° with respect to the plane of the bilayer (Fig. 1D and fig. S1). To define a small area of illumination within the bilayer, a galvo-galvo (GG) scanner and an apodization mask (AM) were conjugated to the back focal plane of the XO. By scanning the focused laser oblique to the bilayer at a higher frequency than acquisition, we created a virtual light sheet that produced a homogeneous illumination field (fig. S2C). The AM contains concentric rings of different diameters that crop the incoming expanded laser to produce a Bessel beam after focusing it into the bilayer (*27*). This creates a smaller and more stable focal point (fig. S2B). Last, a movable mirror (MM) was placed behind the AM to control the angle of illumination (Fig. 1A) by changing the point of illumination on the back focal plane of the XO on the vertical axis. This increased the angle distribution by ±34°.

The light emitted by fluorophores in the bilayer is collected by a commercial upright microscope coupled to a 4-camera splitter (fig. S1). Of the four cameras coupled to the splitter, only three are currently used for FBM experiments, as depicted in Fig. 1. To permit frequent and rapid access to the top chamber, we used water immersion objectives with long WDs. Because SPT requires both sufficient resolution and sufficient emitted light intensity, we found that, along with the focused illumination, the use of a 25× objective lens with a high numerical aperture was critical to detect single molecules at fast frame rates.

### Diffusion of integral membrane proteins

To benchmark the performance of the FBM, we compared the diffusion of the human M2 muscarinic receptor (M_2_R), an archetypical G protein–coupled receptor (GPCR) in our system against total internal reflection fluorescence (TIRF) experiments performed in SBs (*29*). SBs are relatively easy to implement in commercial microscopes, and thus, they are extensively used as lipid membrane models (*30*–*32*).

We formed SBs on three different substrates to account for any variability of the chosen substrate on the system: mica, quartz, and pegylated quartz (*30*, *32*). Fluorescently labeled M_2_R was reconstituted in liposomes of 1,2-dioleoyl-sn-glycero-3-phosphocholine:1-palmitoyl-2-oleoyl-sn-glycero-3-phospho-(1′-rac-glycerol) (DOPC:POPG; 7:3) (*33*). Liposomes were added to the substrate (coverslip) and incubated at 37° to induce bursting and bilayer formation, followed by extensive washing before imaging by TIRF illumination. To perform TIRF experiments, we replaced the FBM experimental chamber with a prism-containing chamber (fig. S3A). The coverslips were then mounted on the prism and imaged using the scanned focused laser of the FBM at total internal reflection (movies S3 to S5). Videos were recorded at 8 Hz. For FBM experiments, we introduced an ALFA-tag at the C terminus of M_2_R to render it amenable to effective and noninvasive fluorescent labeling after fusion to the planar bilayer (*34*). This was done to avoid the increase in background produced by proteoliposomes (PLs) partitioning into the torus. M_2_R was reconstituted in 1-palmitoyl-2-oleoyl-sn-glycero-3-phosphoethanolamine (POPE):POPG (3:1) liposomes and fused into previously formed FBs of the same lipid composition. After fusion, the top chamber was perfused with 5 to 10 chamber volumes to remove unfused vesicles, followed by the addition of 1 nM anti-ALFA nanobody (NbALFA) labeled with LD655. To remove excess NbALFA-LD655 the chamber was perfused with another 10 chamber volumes. The bilayers were then imaged using focused beam illumination, and videos were recorded at 50 Hz (movie S6).

**Fig. 2.**
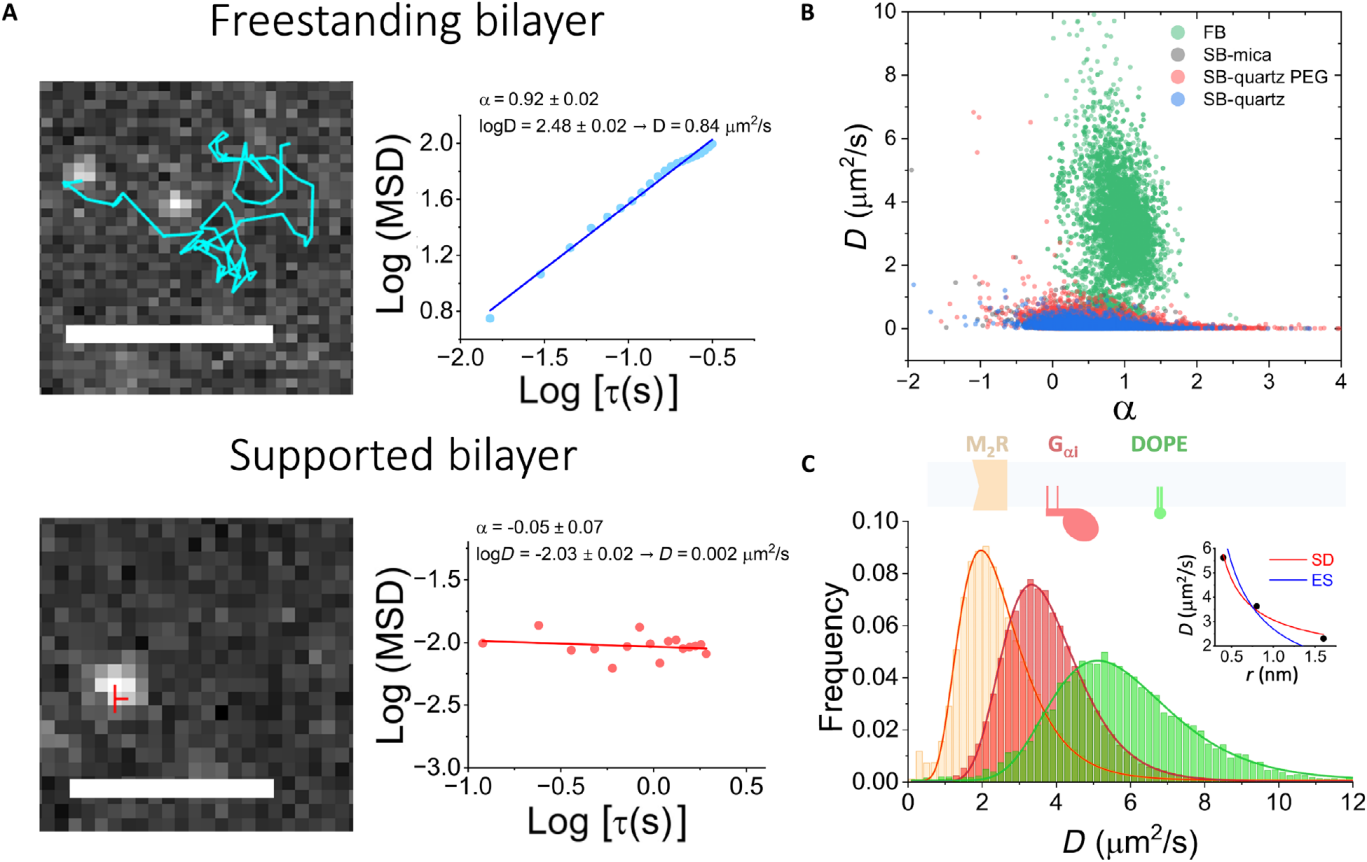
Single-particle tracking benchmark of the FBM. (**A**) Left: Representative SPT data for the M_2_R receptor diffusing in an FB (top) and in a quartz SB (bottom). M_2_R receptor was reconstituted in POPE:POPG (3:1) vesicles for FBM experiments and in DOPC:POPG (7:3) for SB experiments. For FBM experiments, vesicles were allowed to fuse into previously formed POPE:POPG (3:1) bilayers, followed by labeling with LD655-labeled NbALFA and extensive washing. For SBs, M_2_R-containing vesicles were allowed to burst and fuse onto solid substrates followed by washing. M_2_R was labeled with LD655-CoA through AcpS site–directed labeling. SB experiments were performed by TIRF illumination achieved with FBM optics, exchanging the FBM chamber for a prism-containing chamber. Scale bars, (FBM) 5 μm and (SB) 2 μm. Right: Mean squared displacement (MSD) analysis of the tracks on the left. Fit of the data indicates Brownian diffusion only for the FBM (top). (**B**) Diffusion (*D*) versus anomalous diffusion (α) coefficients plots. Data points were obtained from MSD analysis of 7620, 11307, 17731, and 6568 tracks for the FBM (green), mica-supported (gray), quartz-supported (blue), and pegylated quartz–supported (red) membranes. The distribution of points for the FBM experiments (*D* ~ 2 μm^2^/s and α = 1 ± 0.25) indicated that only FBM permits unconstrained diffusion of M_2_R. (**C**) Histrograms of *D* for M_2_R, G_αi1_, and DOPE-Cy5. Experiments were performed using the FBM, observing unconstrained Brownian diffusion of all species. The distributions of *D* were fit to log-normal distributions (*r*^2^ = 0.99) and showed a weak dependence on size. G_αi_ was labeled with LD655 through Sfp site–directed labeling. Inset: Fit of the mean of distributions to a Saffman-Delbrück (SD, *r*^2^ = 0.95) and Einstein-Smoluchowski (ES, *r*^2^ = 0.68) model of diffusion.

Videos for both SB and FBM experiments contained well-resolved diffraction-limited particles that were detected and analyzed using SPT software (*35*). Particle trajectories (tracks) were obtained after particle detection and assignment across successive frames. Figure 2A shows a characteristic example of a track for an FBM (top) and an SB (bottom) experiment. To analyze the tracking data, we plotted the mean squared displacement (MSD) of the track against increasing lag times (τ) and fit the data to the logarithmic anomalous diffusion equation

Log (MSD)=Log(D)+α Log(τ) (1)

where *D* is the generalized diffusion coefficient and α is the anomalous coefficient, which takes a value of 1.0 ± 0.25 for normal (Brownian) diffusion (*10*, *36*). We found that particles in SB are immobile, as evidenced by the shape of the MSD curve and the fitting results (*D* = 0.002 μm^2^/s, α = −0.05). Comparatively, particles in the FB are mobile, and the fit indicates Brownian diffusion (*D* = 0.8 μm^2^/s, α = 0.92). We then performed a similar analysis on thousands of tracks (Fig. 2B). The results, shown as *D* versus α plots, reported a stark difference in the distribution of diffusion and anomalous coefficients; most particles undergo normal diffusion in the FBM, while most particles are immobile in SBs regardless of the substrate material (Fig. 2B and fig. S4A). We reproduced these FBM experiments with two other membrane proteins with different folds and oligomeric structures, the dimer TRAAK and the tetramer GIRK2, finding similar results (fig. S5), i.e., most particles (>80%) underwent Brownian diffusion with diffusivities ~1.0 μm^2^/s.

Unconstrained Brownian diffusion was also observed for phospholipids 1,2-dioleoyl-sn-glycero-3-phosphoethanolamine (DOPE-Cy5) and the fluorescently labeled membrane-associated guanine nucleotide–binding protein G_αi_ (movies S7 and S8). Analysis and classification of the tracks yielded histograms of diffusion coefficients that follow log-normal distributions (*r*^2^ = 0.99) (Fig. 2C). We found that G_αi_ diffuses faster than M_2_R and the lipid DOPE faster than either protein. The relation between the diffusion coefficients of the three species is compatible with a model of diffusion that depends weakly on the hydrodynamic radius. Particularly, our data provide a better fit for a Saffman-Delbrück model (*r*^2^ = 0.95) than for the Einstein-Smoluchowski model (*r*^2^ = 0.68) (Fig. 2C) (*37*).

Besides the substrates tested with SPT, we used fluorescence recovery after photobleaching to examine other solid support preparations (piranha-cleaned quartz and SB formation on top of a Langmuir-Blodgett transferred lipid monolayer or over a transferred bilayer) to explore conditions that would possibly allow free diffusion (*38*). In all cases tested, proteins in SBs were immobilized in contrast to FBs.

We should note that after the fusion of M_2_R-containing liposomes, we observed the presence of large aggregates of protein in the bilayer (fig. S6A). These aggregates have somewhat round shapes and lie in the plane of the bilayer without noticeable height. We have observed similar aggregates for the ion channel GIRK2 (fig. S6B) and TRAAK (see the section “Protein partitioning into phase-separated bilayers”). Proteins exchanged between these aggregates and the rest of the bilayer. They also fused with each other in ways reminiscent of descriptions of protein condensates (fig. S6C). We are currently studying these structures, and their description will not be part of the present publication.

### Applications of the FBM on ion channels: Estimation of open probability with multiple channel recordings

**Fig. 3.**
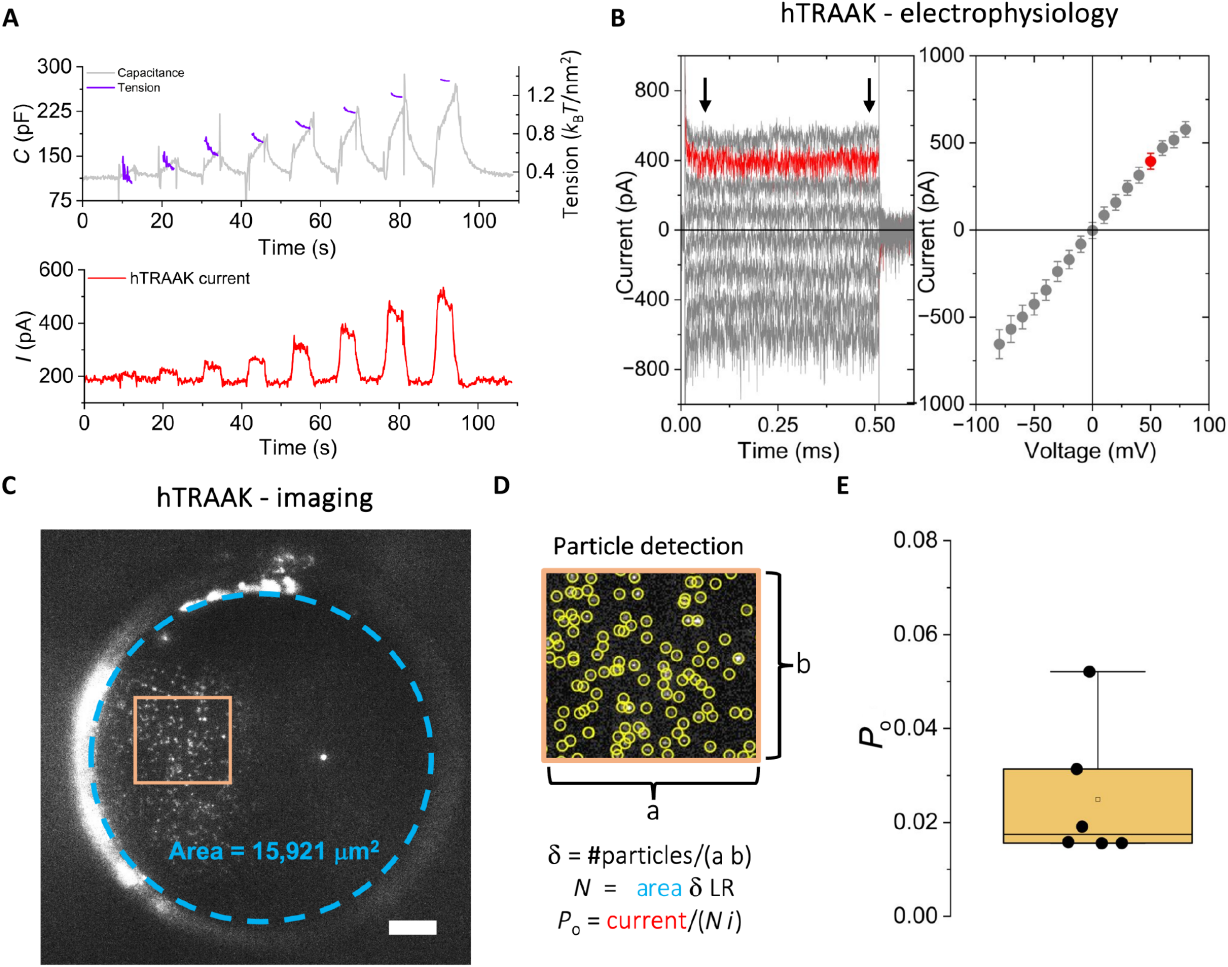
Simultaneous imaging and electrical recordings of ion channels and determination of hTRAAK open probability (*P*_o_). (**A**) Tension activation curves of hTRAAK showing its characteristic mechanosensitivity. The lateral tension of the bilayer (purple) was calculated from changes in the bilayers’ capacitance (gray) upon application of increasing pressure steps from 1 to 8 mmH_2_O (*39*). (**B**) Electrophysiology of hTRAAK. Left: Potassium currents through hTRAAK channels, measured at voltage steps of 500 ms from −80 to 80 mV. Only odd voltage steps are shown for clarity. hTRAAK was reconstituted into POPE:POPG (3:1) vesicles and fused into bilayers of the same composition. Right: Current-voltage characteristic showing the average ± standard deviation of the currents at each voltage. The trace at 50 mV is plotted in red for illustrative purposes, and black arrows indicate the interval for the calculation of the average. (**C**) Imaging of hTRAAK. The frame of a video showing scanned focused laser illumination of the hTRAAK containing bilayer from (A). The dotted blue line shows the perimeter of the bilayer used to calculate its area. Scale bar, 20 μm. (**D**) Detection of hTRAAK tracks analyzed using SPT software. Bottom: Equations used to calculate *P*_o_, where *i* is the single-channel current for hTRAAK and LR is the reciprocal of the labeled fraction of hTRAAK channels. (**E**) *P*_o_ calculation for different bilayers. The data are shown along a box plot showing the median (0.017), 25;75 percentile (box, 0.016;0.031), and 5;95 percentiles (whisker, 0.052) (*N* = 6).

In FB experiments, both sides of the bilayer are isolated, which permits coupling single-particle imaging experiments with functional measurements that require transport across the bilayer. We used this property of the FBM to determine the open probability of an ion channel in bilayers containing multiple channels using simultaneous imaging and electrical recordings. To perform these experiments, we chose the human mechanosensitive potassium channel TRAAK (hTRAAK). We have previously studied hTRAAK in FBs, showing tension sensitivity curves measured with an FB tensiometer that we developed and which is fully compatible with the FBM (*39*) (Fig. 3A).

Determining ion channel open probability (*P*_o_) can usually be accomplished by measuring single channels or performing noise analysis on recordings with multiple channels, so-called macroscopic current recordings (*40*). However, the single-channel kinetics of TRAAK and its very low open probability, which renders a linear relationship between current variance and mean, preclude a good estimation of *P*_o_ by either of the aforementioned methods.

The exact number of channels that contribute to the current is the main unknown in the effort to determine the *P*_o_ of a channel from macroscopic experiments. Using the FBM, we were able to determine the number of hTRAAK channels (*N*) in the bilayer while simultaneously measuring the current through those channels (*I*) (Fig. 3, B to D). Knowing *N* and *I*, we calculated hTRAAK *P*_o_ from the simple relation, *I* = *i N P*_o_, where *i* is the current through a single channel (*41*). We determined the median of the channel *P*_o_ at the bilayer basal tension (0.2 to 0.3 *k*_B_*T*/nm^2^) to be 1.7% (interquartile range = 1.6;3.1) (Fig. 3E). Furthermore, increasing the tension of the bilayer by about 4-fold increased the channel activity by only 2.5-fold.

For these experiments, we purified, labeled, and reconstituted hTRAAK in POPE:POPG (3:1) liposomes. After liposome fusion into FBs of the same lipid composition, we recorded videos of diffusing channels and performed SPT analysis as described above. From this analysis, we calculated the density of detected particles within a region of the bilayer with homogeneous focus and illumination and extrapolated the number of hTRAAK in the entire bilayer after correcting for the labeling efficiency and subsequent multiplying by the total bilayer area (Fig. 3D).

### Protein partitioning into phase-separated bilayers

**Fig. 4.**
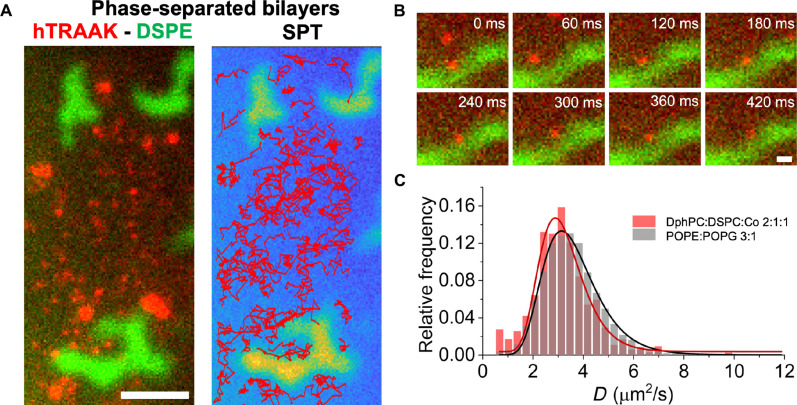
Single-particle tracking of hTRAAK in phase-separated bilayers. (**A**) Representative image of hTRAAK in DphPC:DSPC:cholesterol (2:1:1 molar ratio) Left: The frame from a video overlapping hTRAAK (red) and DSPE-AF488 (green) channels. DSPE partitions into the ordered phase. Right: hTRAAK trajectories obtained from SPT analysis show the preference for hTRAAK for the disordered phase. Scale bar, 10 μm. hTRAAK was labeled with LD655-NHS. (**B**) Sequence of a single hTRAAK diffusing along the boundary between the two phases. Scale bar, 2 μm. (**C**) Diffusion coefficient (*D*) distributions of hTRAAK. *D* was measured by SPT analysis in POPE:POPG (3:1 weight ratio, gray) and in DphPC:DSPC:cholesterol (2: 1:1 molar ratio, red) bilayers showing no apparent difference between the two lipid compositions.

Cell membranes contain hundreds of different kinds of lipids (*42*). It has been shown that the interaction between different species of lipids can give rise to critical phenomena. For example, ternary mixtures of lipids containing a high *T*_m_ phospholipid, a low *T*_m_ phospholipid, and cholesterol can phase-separate in vitro, leading to the coexistence of liquid-ordered and liquid-disordered phases (*43*). In addition, it has been observed that the protein distribution in membranes is not homogeneous, and some have proposed that an inhomogeneous lipid composition could be a driving mechanism of this distribution (*44*). To test whether lipid composition and phase separation can drive an inhomogeneous distribution of proteins, we performed SPT experiments with hTRAAK in bilayers of 1,2-diphytanoyl-sn-glycero-3-phosphocholine (DphPC):1,2-distearoyl-sn-glycero-3-phosphocholine (DSPC):cholesterol (2:1:1), a mixture that phase separates at room temperature, forming liquid ordered and liquid disordered phases (*45*). We added a small amount of fluorescent lipid to label the ordered phase [1,2-distearoyl-sn-glycero-3-phosphoethanolamine-N-(TopFluor AF488), DSPE-AF488]. The data show a notable preference for hTRAAK for the disordered phase (Fig. 4A). This preference is further evidenced by the distribution of tracks that avoid the ordered regions (Fig. 4A, right). TRAAK molecules that approached the boundary between phases often moved along the perimeter before returning to the disordered phase (Fig. 4B). We also found that the diffusion coefficient distribution of TRAAK in the phase-separated bilayer does not differ significantly from the distribution in the binary mixture POPE:POPG (3:1) (Fig. 4C).

## DISCUSSION

IMPs are fundamental elements of cell signaling and energy metabolism. Yet, they remain incredibly difficult to study due to their low expression levels and the complexity of their native environments. Current techniques cannot fully address the dynamics of proteins in membranes; how they interact with themselves and others and how their motion and distribution are affected by the environment.

We introduced FBM, freestanding bilayer microscope, a method to perform in vitro single-molecule imaging of membrane proteins in membranes. We showed that the use of planar FBs, as opposed to SBs, leads to unconstrained free diffusion devoid of artifacts caused by the presence of the substrate. We demonstrated the use of the FBM by performing SPT of the IMP M_2_R, as well as the lipidated G_αi1_ and fluorescent DOPE lipids. From the tracking results, we found that more than 90% of the tracks underwent Brownian diffusion. We determined the distribution of diffusion coefficients for the three species, finding that they fit into a log-normal distribution. By fitting the median of the distributions to the estimated hydrodynamic radius of each molecule, we found that the data correspond better to a Saffman-Delbrück model than to an Einstein-Smoluchowski model, which implies a dependence of diffusion on radius weaker than 1/radius (*37*). In other words, in freestanding membranes, diffusion scales more weakly with protein size than in solution.

The FBM is based on PBs, a proven, effective method for the fully functional reconstitution of different membrane proteins used in different laboratories worldwide. This offers versatility as the FBM setup can easily be combined with other PB-based assays. We demonstrated this by performing simultaneous imaging and electrical recordings of the mechanosensitive channel hTRAAK. hTRAAK is located in the nodes of Ranvier of myelinated neurons of mammals, and it has been shown to play an important role in thermal and mechanical nociception (*46*). Determining the open probability, *P*_o_, of this channel has been challenging owing to its flickery single-channel kinetic behavior. By using the FBM, we determined that the *P*_o_ of hTRAAK at the basal tension of PBs is about 2.5%, with an estimated increase to about 6.3% at tensions up to 1.2 *k*_B_*T*/nm^2^, which is about 60% of the lytic tension needed to rupture a typical lipid membrane. These *P*_o_ values imply that hTRAAK works within a range of low *P*_o_ and are consistent with previous results from our laboratory (*47*).

We also showed that the FBM can be used to study the influence of lipid composition on the diffusion and distribution of membrane proteins. We explored this possibility using a ternary mixture of lipids that phase-separate at room temperature. Incorporation of TRAAK into these bilayers did not change the amount of lipid phase separation (fig. S7), but there was a notable preference of TRAAK for the liquid disordered (*L*_d_) phase. No particles of TRAAK were detected within the ordered phase. This phenomenon suggests a mechanism of exclusion and enrichment of TRAAK that could play an important role in the much more complex plasma membrane of cells.

We anticipate that the FB microscope will be an important tool to elucidate the mechanisms of membrane protein organization and dynamics. For example, the information contained in SPT can be used to study protein-protein interactions in the membrane. Combined with the unique compositional control offered by the planar bilayers, this could lead to new insights into signaling mechanisms that depend on dynamic complex formation, such as GPCRs, receptor tyrosine kinases, or immune receptor pathways. Furthermore, the possibility of combining the FBM with smFRET offers the possibility to study structural changes of proteins as a function of interactions with other species in a reaction-diffusion setting, something that is today only possible with simulations.

Last, our results show that the diffusion of membrane proteins in FBs is different than in cell membranes. Diffusion coefficients in FBs are larger than in cell membranes by about 10-fold, and a larger fraction of the diffusion is Brownian. There are many hypotheses to explain smaller diffusion coefficients, higher immobile fractions, and anomalous diffusion in cell membranes (*4*, *48*, *49*). The FBM provides a good starting point to test these hypotheses experimentally. The FBM is a reductionist system that will permit controlled, stepwise building in complexity so that we may eventually understand the properties of diffusion in cell membranes and, ultimately, the signaling processes that rely on cell membrane diffusion.

## MATERIALS AND METHODS

### M_2_R-ALFA and A1-M_2_R expression and purification

An ALFA-tag was introduced at the C terminus of the human muscarinic 2 receptor (hM_2_R) gene, followed by a 3C PreScission Protease (PPX) recognition site, the enhanced yellow fluorescent protein (eYFP) gene, and a polyhistidine tag (His10-tag) (M_2_R-ALFA). The ALFA-tag was used for binding with an NbALFA conjugated to a fluorophore for single molecule visualization, while the eYFP and His10-tags were inserted for purification purposes (*34*). The gene was then cloned into a pFastBac vector for protein expression with the Bac-to-Bac baculovirus system. *Spodoptera frugiperda* Sf9 insect cells were transfected with isolated bacmid DNA to produce a recombinant baculovirus stock. The virus was amplified through two more rounds of progressive infection of Sf9 cells to create a high-titer stock used for large-scale expression of M_2_R-ALFA. Six liters of Sf9 cells were cultured to a density of 3 million cells/ml and infected with 20 ml of baculovirus stock. The cells were grown for 48 hours after infection at 27°C shaking at 120 rpm, after which cells were pelleted by centrifugation at 3500*g* for 15 min at 4°C. Cell pellets were resuspended in 1× phosphate-buffered saline (1× PBS; Thermo Fisher Scientific) and centrifuged at 3500*g* for 10 min at 4°C. Pellets were flash-frozen in liquid nitrogen and stored at −80°C until use.

A short peptide tag, A1, was cloned at the N terminus of the hM_2_R gene to enable post-purification AcpS phosphopantetheinyl transferase (PPTase) catalyzed site-specific protein labeling (A1-M_2_R) (*50*). At the C terminus of the hM_2_R gene, the PPX recognition site, eYFP gene, and His10-tag were included for purification purposes. A1-M_2_R was expressed as described for M_2_R-ALFA.

M_2_R constructs (M_2_R-ALFA and A1-M_2_R) were purified following the same protocol (*51*). All purification steps were carried out at 4°C unless specified otherwise. Sf9 cells were lysed by osmotic shock by resuspending the pellets in the following buffer for 30 min: 10 mM tris-HCl (pH 7.4), 10 mM MgCl_2_, benzonase nuclease (5 U/ml), 5 mM β-mercaptoethanol (βME), 2 mM phenylmethylsulfonyl fluoride (PMSF), 1× aprotinin saline solution [3 to 7 trypsin inhibitor units (TIU)/mg], and 0.1% v/v of protease inhibitor cocktail (PIC) stock [leupeptin (1 mg/ml), pepstatin A (1 mg/ml), and 1 M benzamidine HCl]. After mixing for 30 min, the lysis slurry was centrifuged at 30,000*g* for 20 min. Once the supernatant was removed, cell pellets were resuspended in high salt buffer [20 mM Hepes (pH 7.4), 750 mM NaCl, 10% v/v glycerol, 5 mM βME, benzonase nuclease (5 U/ml), 5 μM atropine, 1× aprotinin saline solution (3 to 7 TIU/mg), and 0.1% v/v of PIC stock] and homogenized using a Dounce homogenizer. IMPs were extracted by solubilizing the membrane with 1%:0.05% *n*-dodecyl-β-d-maltopyranoside:cholesterol hemisuccinate (DDM:CHS) for 1 hour at room temperature, followed by 1 hour at 4°C. After extraction, insoluble material was pelleted by centrifugation at 30,000*g* for 30 min. The supernatant was bound to TALON resin and washed with buffers [20 mM Hepes (pH 7.4), 0.1%:0.01% DDM:CHS, 1 μM atropine, 1 mM βME, and 0.1% v/v of PIC stock] containing a gradual increase in NaCl concentration. The resin was washed with five column volumes (CVs), referring to the volume of resin used, of wash buffer containing 750 and 587 mM NaCl, respectively. Next, the resin was washed with five CVs of wash buffer with 424 mM NaCl and 20 mM imidazole. M_2_R was eluted with 261 mM NaCl and 200 mM imidazole wash buffer.

The elution fractions were pooled and subsequently diluted tenfold with the following dilution buffer to lower the imidazole concentration and introduce the superagonist iperoxo: 20 mM Hepes (pH 7.4), 100 mM NaCl, 0.1%:0.01% DDM:CHS, 0.1 mM Tris(2-carboxyethyl)phosphine (TCEP), and 12 μM iperoxo. The protein was then bound to anti_GFP nanobody–coupled Sepharose resin (GFPNb resin), produced in-house as previously described, for 1 hour (*51*). The protein was buffer exchanged by washing the resin on the gravity column with 20 CVs of buffer containing the same components as the dilution buffer, except for an increased iperoxo concentration of 50 μM. PPX was added to the resin. The protein was nutated overnight to ensure complete digestion and to change the ligand bound to M_2_R from the antagonist atropine to iperoxo. The cleaved M_2_R was eluted by collecting the flow through of the GFPNb resin by gravity.

A1-M_2_R was labeled with twofold excess LD655-CoA dye in the presence of 10 mM MgCl_2_, 2 mM TCEP, and a 1:5 ratio of AcpS (expressed and purified in-house) to A1-M_2_R. The enzyme-catalyzed fluorophore conjugation reaction was allowed to proceed in the dark for 2 hours at room temperature, followed by 2 hours at 4°C. A labeling efficiency of ~10% was attained using this method. M_2_R-ALFA was fluorescently labeled through binding with NbALFA-LD655 after vesicle fusion into FBs.

M_2_R constructs were dephosphorylated through a 30-min room-temperature treatment with 200 U of lambda protein phosphatase (NEB), 10 U of calf intestinal phosphatase (NEB), 5 U of antarctic phosphatase (NEB), and 1 mM manganese chloride. Proteins were concentrated in 30-kDa molecular weight cutoff (MWCO) concentrators before their respective purification by size exclusion chromatography using a Superdex 200 Increase 10/300 GL column and running buffer [20 mM Hepes (pH 7.4), 100 mM KCl, 50 mM NaCl, 10 μM iperoxo, 100 μM TCEP, and 0.1%:0.01% DDM:CHS]. M_2_R reconstitution in lipid vesicles was done immediately after gel filtration. Purified M_2_R that was not reconstituted was flash-frozen with 10% glycerol and stored at −80°C.

### G_αi1_ expression and purification

The short peptide tag, S6, was inserted in an internal loop between amino acids 112 and 113 of the human G_ɑi1_ gene (*50*). The S6 tag was inserted for site-specific protein labeling via the Sfp PPTase enzyme–catalyzed peptide tag modification. The G_ɑi1_-S6 gene was cloned into the pFastBac vector. Genes for the wild-type human G_β1_ and G_γ2_ subunits were also cloned into the pFastBac vector. At the N terminus of G_γ2_, a His10-eYFP linked to a PPX recognition site was inserted. Recombinant baculovirus for each subunit was prepared separately as described above. For large-scale expression of the heterotrimeric G protein, 6 liters of *Trichoplusia ni* High Five insect cells were cultured to a density of 2 million cells/ml. Each liter of cells was coinfected with an experimentally determined ratio of G_ɑi1_, G_β1_, and G_γ2_ baculovirus (8 ml of G_γ2_, 12 ml of G_β1_, and 15 ml of G_ɑi1_) to achieve expression of the heterotrimeric G protein subunits in stoichiometric proportions. The cells were grown for 36 to 48 hours and then harvested by centrifugation at 3500*g* for 15 min at 4°C. Cell pellets were resuspended in 1× PBS and centrifuged at 3500*g* for 10 min. Pellets were flash-frozen and stored at −80°C until further use.

All purification steps were carried out at 4°C unless specified, and stock guanosine 5′-diphosphate (GDP) disodium salt aliquots were made fresh before use. Cell pellets were resuspended in buffer containing 20 mM tris-HCl (pH 8), 100 mM NaCl, 5 mM dithiothreitol (DTT), 3 mM MgCl_2_, 20 μM GDP, 2 mM PMSF, deoxyribonuclease (DNase) I, 1× aprotinin saline solution (3 to 7 TIU/mg), and 0.1% v/v of PIC stock. After mixing for 15 min, the cell slurry was manually homogenized. Membranes were extracted in 1% sodium cholate for 1.5 hours. The extraction was centrifuged at 35,000*g* for 1 hour. The supernatant was bound to GFPNb resin and then washed with 10 CVs of wash buffer [20 mM tris-HCl (pH 8), 100 mM NaCl, 1% Na-cholate, 3 mM MgCl_2_, and 20 μM GDP] on the column. G_ɑi_ elution buffer [20 mM tris-HCl (pH 8), 100 mM NaCl, 1% Na-cholate, 50 mM MgCl_2_, 30 μM GDP, 10 mM NaF, and 30 μM AlCl_3_] was used to elute G_ɑi1_-S6 from the G_βγ_ complex bound to the GFPNb resin. The resin was moved to room temperature, and the elution buffer was warmed in a 30°C water bath for 15 min; GDP was added to the elution buffer afterward. The resin and elution buffer were incubated together, nutating for 30 min at room temperature, and G_ɑi1_-S6 alone was collected by gravity flow. The eluate containing G_ɑi1_-S6 was labeled using twofold molar excess LD655-CoA dye, Sfp enzyme that was purified in-house (1:2 ratio of enzyme to protein), and 10 mM MgCl_2_. A labeling efficiency of ~10% was obtained. G_ɑi1_ was dephosphorylated through a 30-min room-temperature treatment with 200 U of lipid phosphate phosphohydrolase, 10 U of calf intestinal phosphatase, 5 U of alkaline phosphatase, and 1 mM manganese chloride. The protein was concentrated and purified using a Superdex 200 Increase 10/300 GL column [20 mM tris-HCl (pH 7), 150 mM KCl, 10 mM DTT, 0.4 mM TCEP, 1% Na-cholate, 2 mM MgCl_2_, and 20 μM GDP]. Fractions containing LD655-G_ɑi_ were collected and reconstituted into lipid vesicles. Non-reconstituted protein was flash-frozen with 10% glycerol and stored at −80°C.

### hTRAAK expression and purification

The human mechanosensitive potassium channel TRAAK (hTRAAK) gene was cloned into a modified pPICZ-B vector to create a fusion protein construct containing a C-terminal PPX cleavage site, the GFP gene, and His10-tag. The hTRAAK protein construct was then transformed into *Pichia pastoris* strain SMD11-63H and expressed in large-scale cultures as described previously (*52*). Cell pellets were flash-frozen in liquid nitrogen and subsequently disrupted by milling five times for 3 min at 25 Hz. Lysed cells were frozen and stored at −80°C until they were ready for purification.

All purification steps were carried out at 4°C. The lysed cells were mixed with a fivefold excess volume of buffer [50 mM tris-HCl (pH 8.5), 150 mM KCl, 1 mM EDTA, DNase I, 1× aprotinin saline solution (3 to 7 TIU/mg), and 0.1% v/v of PIC stock] relative to the wet pellet volume for 15 min. The membranes were manually homogenized and extracted using 2% DDM for 1.5 hours. The extraction mixture was centrifuged at 39,000*g* for 30 min. The supernatant was bound to GFPNb resin for 1 hour. The resin was washed with 10 CVs of wash buffer [20 mM Hepes (pH 8), 150 mM KCl, and 0.1% DDM]. PPX was added to the resin in a 1:20 protease-to-protein ratio, and the protein was digested overnight. The flow through containing cleaved TRAAK was collected from the resin and was concentrated using a 10-kDa MWCO concentrator.

TRAAK was nonspecifically labeled using amine-reactive LD655-NHS dye. The protein was buffer exchanged using a PD-10 column into 1× PBS without calcium and magnesium at pH 8. Fifty-fold molar excess LD655-NHS dye was added to the protein solution; protected from light, the reaction was allowed to proceed overnight at 4°C. The protein was labeled with an efficiency of 15%. Labeled TRAAK was concentrated using a 10-kDa MWCO concentrator and subsequently purified using a Superdex 200 Increase 10/300 GL column [20 mM tris-HCl (pH 7.4), 150 mM KCl, 1 mM EDTA, and 0.03% DDM]. Fractions containing LD655-TRAAK were reconstituted.

### GIRK-ALFA expression and purification

At the C terminus of hGIRK2, a PPX cleavage site, eGFP gene, and His10 tag were preceded by an ALFA-tag. The fusion protein construct was cloned into the pBacMam vector and the BacMam system was used to create a high titer recombinant baculovirus stock using Sf9 cells. Four liters of the HEK293S GnTI^−^ strain from ATCC (CRL-3022) were cultured to a density of 3.5 million cells/ml and infected with 100 ml of the high titer GIRK2-ALFA recombinant baculovirus. After 12 hours, the cells were induced with 10 mM sodium butyrate. After shaking at 37°C for an additional 40 to 48 hours, the cells were harvested by centrifugation at 3500*g* for 15 min at 4°C. Cell pellets were resuspended in 1× PBS and centrifuged at 3500*g* for 10 min at 4°C. The supernatant was discarded. Cell pellets were flash-frozen and stored at −80°C.

Purification of GIRK2-ALFA was carried out at 4°C unless noted otherwise. Cell pellets were first mixed in resuspension buffer [25 mM tris-HCl (pH 7.5), 150 mM KCl, 50 mM NaCl, 1 mM MgCl_2_, 1 mM CaCl_2_, 2 mM DTT, 2 mM PMSF, DNase I, 1× aprotinin saline solution (3 to 7 TIU/mg), and 0.1% v/v of PIC stock] for 15 min. The cell slurry was homogenized (Dounce), and the lysate was centrifuged at 39,000*g* for 15 min. The supernatant was discarded. Pellets were suspended in buffer [25 mM tris-HCl (pH 7.5), 150 mM KCl, 50 mM NaCl, 1 mM MgCl_2_, 1 mM CaCl_2_, 2 mM DTT, 1× aprotinin saline solution (3 to 7 TIU/mg), and 0.1% v/v of PIC stock] and homogenized (Dounce). Membranes were extracted with 1.5%:0.3% DDM:CHS for 2 hours. The extraction was centrifuged at 39,000*g* for 30 min to pellet the insoluble fraction, and the supernatant was bound to GFPNb resin for 1 hour. The resin was washed on a column with 20 CVs of wash buffer [20 mM tris-HCl (pH 7.5), 150 mM KCl, 50 mM NaCl, 2 mM DTT, and 0.05%:0.01% DDM:CHS]. PPX was added to the resin slurry for overnight digestion. The flow through containing cleaved GIRK2-ALFA was collected from the GFPNb resin by gravity flow. The protein was concentrated using a 100-kDa MWCO concentrator and purified of contaminants using a Superose 6 Increase 10/300 GL column with the buffer: 20 mM tris-HCl (pH 7.5), 150 mM KCl, 50 mM NaCl, 10 mM DTT, 1 mM Na_2_EDTA (pH 7.4), and 0.025%:0.005% DDM:CHS. GIRK2-ALFA was reconstituted into liposomes after gel filtration. The GIRK2-ALFA construct was fluorescently labeled with NbALFA-LD655 after vesicle fusion into FBs.

### Anti-ALFA nanobody expression and purification

The NbALFA was expressed as an N-terminal His14-SUMO fusion in *Escherichia coli* cells (*34*). The recombinant plasmid was transformed into One Shot BL21 Star (DE3) cells. A single colony was used to inoculate small-scale cultures: 50 ml of lysogeny broth (LB) with 50 μg/ml of kanamycin. The cells were grown overnight (~18 hours) at 37°C shaking at 225 rpm. The next morning, 20 ml of small-scale culture was added to 1 liter of LB supplemented with kanamycin (50 μg/ml) for a 1:50 dilution. Once the cells reached an optical density (OD) at 600 nm of about 0.6, the cells were induced with 0.5 mM isopropyl-β-d-thiogalactopyranoside and allowed to grow overnight at 16°C. Cells were harvested by centrifuging at 3500*g* for 15 min at 4°C. Cell pellets were resuspended in 1× PBS, and centrifuged at 3500*g* for 10 min at 4°C. Flash-frozen pellets were stored at −80°C.

All purification steps were carried out at 4°C unless otherwise specified. A cell pellet was suspended in lysis buffer {20 mM Hepes (pH 7.9), 300 mM NaCl, 2 mM PMSF, DNase I, 1 mM TCEP, 2× aprotinin saline solution (3 to 7 TIU/mg), and 0.2% v/v of a protease inhibitor mixture stock [trypsin inhibitor (0.1 g/ml), pepstatin A (1 mg/ml), leupeptin (1 mg/ml), 1 M benzamidine HCl, and 0.5 M AEBSF]} and mixed for 15 min. The cells were lysed by sonication and the lysate was clarified by centrifuging at 16,500 rpm for 40 min at 4°C. The supernatant was bound to equilibrated nickel–nitrilotriacetic acid (Ni-NTA) resin for 1 hour and washed with wash buffer [20 mM Hepes (pH 7.9), 300 mM NaCl, and 1 mM TCEP] containing no imidazole followed by 20 mM imidazole. ALFANb was eluted off the resin with 400 mM imidazole-containing wash buffer. The His14-SUMO tag was cleaved of the protein by adding ULP1 (prepared in-house) to the elution. The NbALFA protease solution was placed in 8-kDa MWCO dialysis tubing and dialyzed overnight at room temperature against a buffer composed of 20 mM Hepes (pH 7.9), 300 mM NaCl, 0.5 mM TCEP, and 2 mM DTT. The insoluble precipitate was removed by centrifuging the protein for 10 min at 3500*g*. The supernatant was run through Ni-NTA resin equilibrated in a wash buffer containing 10 mM imidazole to collect cleaved NbALFA. The digested protein was concentrated with a 10-kDa MWCO concentrator and purified using a Superdex 75 10/300 GL column [20 mM Hepes (pH 7.9), 150 mM NaCl, and 1 mM TCEP]. Purified NbALFA was stored at −80°C.

To prepare fluorescent NbALFA for binding ALFA-tagged IMPs on the bilayer, the cysteines of purified NbALFA were nonspecifically labeled with maleimide LD655 (LD655-MAL). Thawed NbALFA was incubated with 0.1 mM of freshly made TCEP at room temperature for 10 min. The reduced protein was buffer exchanged into 1× PBS (not containing calcium and magnesium) at pH 7 to 7.5 using a PD-10 desalting column to remove excess TCEP, which has been found to interfere with high-efficiency bioconjugation by reacting with maleimides to form other products. The NbALFA was mixed with fivefold molar excess LD655-MAL and reacted overnight in the dark at 4°C. To separate the labeled nanobody from excess dye, the protein was run on the Superdex 75 10/300 GL column equilibrated with 20 mM Hepes (pH 7) and 150 mM NaCl. A labeling efficiency of ~80% was achieved; NbALFA-LD655 was stored at −80°C.

### PLs reconstitution

Membrane proteins were reconstituted into liposomes immediately after labeling (if applicable) and purification. A given protein was reconstituted using a specific mixture of phospholipids. The following general protocol to dry lipid films was repeated for all lipid combinations used to reconstitute the proteins discussed in this paper. The desired ratio of lipids in chloroform was mixed in a glass vial, and the chloroform was evaporated under a steady stream of argon gas. The lipid film was solubilized in a small amount of pentane and the solvent was again evaporated under a steady stream of argon. The lipid film was thoroughly dried by storage in a vacuum desiccator overnight.

For FBM experiments, M_2_R-ALFA was reconstituted into a 3:1 weight ratio of POPE:POPG lipids. After removal from the desiccator, the lipid film was rehydrated in buffer [20 mM Hepes (pH 7.4), 100 mM KCl, 50 mM NaCl, 10 μM iperoxo, and 100 μM TCEP] to a stock concentration of 10 mg/ml. The POPE:POPG (3:1) lipids were sonicated to clarity in a water bath sonicator. One percent *n*-decyl-β-maltoside (DM) was added to the lipids to foster protein insertion into vesicles. The lipids were nutated for 30 min at room temperature and sonicated again. To create a protein-to-lipid ratio (PLR) of 1:20, M_2_R-ALFA and POPE:POPG lipids were combined to a final working concentration of 0.25 and 5 mg/ml, respectively. The mixture was nutated for 1 hour at room temperature. To remove the detergent, biobeads (Bio-Beads SM-2 Adsorbent, Bio-Rad) equilibrated in 20 mM Hepes (pH 7.4), 100 mM KCl, 50 mM NaCl, 10 μM iperoxo, and 100 μM TCEP were added to the protein-lipid mixture so that the dry volume of beads was roughly one-third the volume of vesicles. The mixture was moved to nutate at 4°C and biobeads were changed every 8 to 12 hours for a total of three to four changes. After detergent removal, the PLs were harvested, flash-frozen, and stored at −80°C.

A1-M_2_R was reconstituted into a 7:3 weight ratio of DOPC:POPG lipids for SB experiments. The same reconstitution process and buffers were used as described above for M_2_R-ALFA. The protein was protected from light during reconstitution to prevent bleaching of the LD655 fluorophore conjugated to the receptor.

G_ɑi1_-S6 was reconstituted into a 3:1 weight ratio of POPE:POPG lipids. The lipids were brought to a concentration of 10 mg/ml using 20 mM tris-HCl (pH 8), 150 mM KCl, and 2 mM MgCl_2_ and sonicated to clarity in a water bath sonicator, followed by the addition of 1% DM and room-temperature incubation for 30 min. After more sonication, a mixture with G_ɑi1_-S6 (0.405 mg/ml) and lipids (5 mg/ml) was made for a PLR of 1:12. The mixture was nutated for 1 hour at room temperature in the dark. Dialysis was then used to remove the detergent. The mixture was placed in 10-kDa MWCO dialysis tubing, and the buffer [20 mM tris-HCl (pH 8), 150 mM KCl, 20 mM MgCl_2_, and 5 mM DTT] was exchanged five times every 12 hours, adding fresh DTT each time. PLs were harvested, flash-frozen, and stored at −80°C.

For FBM experiments, GIRK2-ALFA was reconstituted into a 3:1 weight ratio of POPE:POPG lipids. Lipids dried overnight were made 20 mg/ml using buffer [20 mM tris-HCl (pH 7.5), 150 mM KCl, and 50 mM NaCl]. Lipids were sonicated to clarity, and 1% DM was added. After lipids were incubated at room temperature for 30 min, they were sonicated again and combined with GIRK2-ALFA (2 mg/ml) in equal parts to create a final PLR of 1:10. Following a 1-hour nutation at room temperature, the vesicles were placed in dialysis tubing of 50-kDa MWCO and dialyzed in 10 mM K_2_HPO_4_ (pH 7.4), 150 mM KCl, and 1 mM K_2_EDTA. The buffer was exchanged five times every 12 hours, adding fresh DTT for each change. For the last two buffer exchanges, equilibrated biobeads were included in the buffer. PLs were aliquoted, flash-frozen, and stored at −80°C.

TRAAK was also reconstituted into a 3:1 weight ratio of POPE:POPG. Lipids were dissolved at 10 mg/ml in 20 mM tris-HCl (pH 7.4) and 150 mM KCl buffer. The lipids were sonicated to clarity. One percent DM was added, and the lipid mixture was nutated at room temperature for 30 min, followed by further sonication. A PLR of 1:10 was achieved by combining channel and lipids for a final concentration of 0.5 and 5 mg/ml, respectively. After 1 hour of incubation at room temperature, the vesicles were dialyzed in 30-kDa MWCO tubing using 10 mM tris-HCl (pH 7.4) and 150 mM KCl buffer containing equilibrated biobeads. The buffer was exchanged every 12 hours for a total of three times. The vesicles were then removed from dialysis tubing and mixed directly with biobeads for 6 hours before being harvested, flash-frozen, and kept at −80°C until use.

### FBM setup: Detection optics

Data acquisition was done with up to four scientific complementary metal-oxide semiconductor cameras (Orca-Flash4.0 V3, Hamamatsu) connected to a commercial upright microscope (FN1, Nikon Instruments), with four 10-mm spacers between the arm and the head (FN-S10, Nikon Instruments) through a camera splitter (Multicam LS image splitter, Cairn research) (fig. S1). The camera splitter was equipped with emission filters (ET460/50 m, ET525/50 m, ET605/70 m, and ET700/75 m, Chroma Technology) and dichroic mirrors (T495LPXR, T565LPXR, and T660LPXR, Chroma Technology) for simultaneous fluorescent recordings of different fluorophores. Cameras were connected to a workstation (Dual Xenon 8-core, 128 GB of memory, a Titan XP video card, and 2 hard drives, 4× NVME 1 TB, and 8× HDD 8 TB) through a Camera Link communication protocol (V3 Firebird Camlink Board). Data acquisition and storage were done with commercial software (NIS-Elements AR 5.11, Nikon Instruments). For transmitted light, a manually controlled quartz halogen lamp was housed in the microscope (FN-LH, Nikon Instruments) (Fig. 1D). For epifluorescence measurements, a tunable light source (SPECTRA X Light engine, Lumencor) with excitation filters (395/25, 440/20,270/24, 510/25, 550/15, 575/25, and 640/30, Chroma Technology) was attached to the microscope head and controlled by computer through the Nis-Elements software. For all the experiments shown in this work, a 25×, 1.1 numerical aperture (NA), 2-mm WD objective was used (CFI75, Apochromat Multi-Photon LWD 25×, Water immersion, Nikon Instruments).

### FBM setup: Excitation optics (focused laser illumination)

For the focused laser illumination, three solid-state continuous wave lasers (473-, 561-, and 660-nm GEM lasers, Novanta Photonics) were expanded using pairs of convergent lenses in a Keplerian design (C240TME-A, 8 mm focal length, AC127-025-AB, 25 mm focal length, Thorlabs) (BE in fig. S1), and combined, using an array of dichroic (T495lpxr, T600lpxr, Chroma Technologies), broadband dielectric mirrors (BB111-E01, Thorlabs), and right-angle mirrors (MRA10-E02, Thorlabs) mounted on a custom-made support (BC in fig. S1). The combined beam was fed into a 4-mm GG scanner (LSKGG4/M, Thorlabs) (Fig. 1D and fig. S1). A laser shutter was placed before the GG scan head for fast laser gating (LS6, Uniblitz Electronics). The lasers were operated by SMD12 PSU (Novanta Photonics) controllers connected to the workstation through a PXI serial interface module (PXI-8430/4, National Instruments) housed in a PXI chassis (NI PXI-1033, National Instruments). The GG scan head was, in turn, operated by a GG controller (Thorlabs) driven by a user-defined signal (DC or sine wave) through a BNC analog output (BNC-2110, National Instruments) also housed in the PXI chassis by an analog output module (PXI-6723, National Instruments). An in-house written software in LabView (National Instruments) was used to control the lasers, the shutter, and the GG. The output of the GG was conjugated to an AM (Annular Mask, Photo Sciences) (*53*), which was itself conjugated to the back focal plane of the XO (TL20X-MPL, 20X, 0.6NA, 5.5 WD, Thorlabs) by relay optics (L1 to L3) using a 4f configuration. L1 (f_1_ = 75 mm, f_2_ = 100 mm, Thorlabs), L2 (f_1_ = 150 mm, f_2_ = 100 mm, Thorlabs), and L3 (f_1_ = 125 mm, f_2_ = 125 mm, Thorlabs) were made with 30-mm cage components (Thorlabs) mounted on custom-made adaptor posts for the case of L1 and adaptor plates for L2 and L3. L2 and L3 adaptor plates were, in turn, mounted on a modified breadboard (MB2530/M, Thorlabs) designed to raise and orient the plane of excitation optics orthogonal to the detection optics plane (fig. S1). All custom-made parts, as well as the optomechanical models of the microscope, were designed using computer-assisted design software (Inventor Profesional, AutoDesk). All machined parts were fabricated in Aluminum 6061 by a manufacturing service (Xometry) following our designs.

### FBM setup: Experimental chamber

The design of the experimental chamber was optimized through many iterations of experimental tests, both imaging and electrophysiology. The chamber (Fig. 1B) was 3D-printed in-house using a MultiJet printer (ProJet MJP 3600, 3D Systems) on a proprietary resin (VisiJet M3 Crystal, 3D Systems). The chamber was designed to contain the XO through a conically shaped aperture (Fig. 1B and fig. S3B) covered by a silicon rubber film to prevent leakages (high-purity high-temperature silicone rubber sheet, 1/32″ thickness, 50A durometer, McMaster-Carr) perforated at the center with a 3-mm punch, to allow the access of the XO. To translate the chamber in space with the 1-μm resolution, it was screwed through two M6 screws (Fig. 1 and figs. S1A and S3B) to a right-angle adaptor plate (3DMS-3X3, Sutter Instruments) mounted on a micromanipulator (MP-285, Sutter Instruments). Parallel to the detection optics plane, two access ports were made to allow for buffer perfusion and pressure regulation (Fig. 1) using a hydrostatic manometer made from a modified 30-ml syringe and another micromanipulator (fig. S1). For all the experiments shown in this work, no bottom perfusion was done, and the chamber was connected solely to the manometer. A three-way valve was added to the tubing connecting the chamber and the manometer to introduce an Ag/AgCl electrode when performing electrophysiological experiments. The top part of the chamber was coupled to the sample holders (cups), which were also 3D-printed in-house by MultiJet printing (Fig. 1B). The mounting was done with vacuum grease (high vacuum grease, Dow Corning) to prevent leaks during electrical measurements. The center of the cup was aligned to a cylindrical hole traversing the entire chamber used for transmitted illumination (fig. S3B). The bottom of the chamber was sealed with a glass coverslip. For the perfusion of solution on top of the cup, two perfusion lines were mounted with wax on the chamber providing access to the cup (Fig. 1B). These lines were connected to a custom-made manual perfusion system.

### Electrophysiology setup

For electrical recordings, the voltage across the lipid bilayer was clamped with an amplifier in whole-cell mode (Axopatch 200B, Axon Instruments) by two Ag/AgCl electrodes placed on the cup and in the manometer line (see the previous section). The analog current signal was filtered at 1 kHz (low-pass, Bessel) and digitized at 10 kHz (Digidata 1550B digitizer, Molecular Devices). Digitized data were recorded on a computer using the software pClamp (Molecular Devices) and analyzed using Clampfit (Molecular Devices).

### FEP partition fabrication

FEP sheets (FEP Copolymer Film, 0.075 mm, Goodfellow) were cut into 1 × 1 cm^2^ and indented at the center, either with a weak CO_2_ laser or a tungsten needle. By sparking the partitions between a high-frequency generator (BD10AS, Electro-Technic Products) and a sharp needle, the damaged region on the partition melted, leaving a round and smooth hole (Fig. 1A). Changing the duration and intensity of the spark led to hole sizes ranging from 100 to 500 mm. Perforated partitions were sonicated for 30 s and stored in ethanol for up to 5 days.

### PB formation

Before FBM experiments, a small amount (0.5 μl) of lipid solution (10 mg/ml in decane) was added to both sides of the partition and left to dry for ~10 min. After mounting a cup on the experimental chamber, the entire chamber was filled with imaging buffer [IB; 150 mM KCl, 10 mM Hepes (pH 7.4), 2.5 mM protocatechuic acid, and 5 nM protocatechuic acid dehydrogenase] followed by the addition of the perfusion adaptors. The perfusion lines were purged of air by perfusing 1 to 2 ml of IB, after which the system was allowed to equilibrate for 5 min. Then, FEP partitions were carefully mounted onto the cups with vacuum grease, and the top electrode was inserted into the top chamber, leading to a sharp increase in current. PBs were then made by applying a small amount (1 to 10 nl) of lipids across the hole of the bilayer. To do this, 1 μl of lipid was first loaded on a pipette and then quickly ejected into the air, leaving a residual lipid solution on the pipette. The pipettes were then inserted into the buffer on the cup, and the bilayer was formed by pushing an air bubble on top of the partition’s hole. The formation of the bilayer was clear from imaging (Fig. 1A) and electrical recordings (quick reduction in the current followed by a progressive increase in capacitance). All experiments were done at room temperature. Experiments were done using POPE:POPG (3:1 weight ratio) lipids, except for the phase separation experiments.

### PL fusion

Unless noted otherwise, PLs containing the IMPs of interest were mixed with an equal volume of 1 M KCl, briefly sonicated (1 s), added on top of the bilayer, and left to sink for 2 min before adding 1 μl of 3 M KCl to induce the fusion of vesicles on the bilayer. Unfused PLs were washed away by perfusing 5 ml of IB. For PLs containing G_αi1_, KCl induction was not necessary as we found that G proteins can diffuse from liposomes into the FB.

### Imaging with the FBM

Focused laser illumination was turned on after perfusion for hTRAAK and G_αi1_, while for M_2_R and GIRK2, illumination was preceded by the addition of 1 nM labeled NbALFA and another 5 ml of perfusion. The illuminated area was adjusted for every bilayer by changing the input voltage amplitude on the GG. To create a square illumination, two sine waves (10 to 100 mV) were used, one for the horizontal and one for the vertical scan, at frequencies of 400 and 2500 Hz, respectively (fig. S2C). Videos were recorded, typically at a frame rate of 50 Hz and with a bit depth of 12-bit (12-bit sensitive mode in Nis-Elements 5.11). SPT was done using uTrack software to obtain the diffusion coefficient (*D*) and the anomalous coefficient (α) for every track (*35*). Further analysis of the data was done using custom-written software in MATLAB (MathWorks). *D* obtained from the analysis (in pixels^2^ per frame) was converted to square micrometers per second using the effective pixel size (0.26 μm for the 25× objective) and the time difference between consecutive frames.

Analysis of the tracks (fig. S4A) to identify the fraction of immobile particles was done by selecting a diffusion cutoff based on the 1% lowest diffusion coefficients (0.5 μm^2^/s) of the distribution for M_2_R measured with the FBM. Particles with diffusion coefficients (*D*) lower than this threshold were considered immobile, while particles with higher *D* were considered Brownian. Particles with an anomalous coefficient (α) higher than 1.25 were considered directed. Brownian and confined-Brownian were not distinguished in this analysis. To analyze the single tracks for Fig. 2A, videos were cropped around a particle, and SPT was performed using TrackMate (Fiji) (*54*). Coordinates were then extracted to calculate the mean squared displacement for different increments of time (MSD versus τ) and then fitted using Eq. 1.

### Quartz coverslip preparation

For quartz SB experiments, quartz coverslips were plasma-cleaned with oxygen as a processing gas for 0.5 to 1 min. Pegylation of quartz coverslips was done as explained in (*55*). Briefly, quartz coverslips were cleaned by dipping them in different cleaning solutions, in the following order: acetone, 1 M KOH, and piranha (H_2_SO_4_:H_2_O_2_ 3:1), followed by washes with deionized water. After cleaning, coverslips were placed in a staining jar with 100 ml of methanol, 5 ml of acetic acid, and 3 ml of 3-aminopropyltriethoxysilane. After 30 min, the solution was replaced by fresh methanol, and this was repeated three times. The coverslips were then dried with Nitrogen gas and incubated with a 0.1 M sodium bicarbonate (pH 8.5) solution with 0.6 mM biotinylated NHS-ester PEG (5000 Da) and 25 mM NHS-ester mPEG (5000 Da) for 3 to 5 hours. Last, coverslips were rinsed with deionized water, dried with nitrogen gas, and stored at −20°C until use. We should note that pegylated quartz coverslips showed fewer fluorescent particles than the other methods, implying that pegylation prevented SB formation to some degree (movies S3 to S5).

### Mica coverslip preparation

Mica coverslips were prepared by coupling a mica sheet to a quartz coverslip with optical glue as described elsewhere (*56*). Briefly, coverslips were cleaned with 2% detergent (Hellmanex III, Hellma Analytics) and ethanol before gluing previously cut mica leaflets (5 mm × 5 mm, 1872-CA, SPI) on top of them with a low viscosity optical adhesive (NOA60, Norland Products). After curing the adhesive with 1-hour exposure to ultraviolet (UV) light, another coverslip was glued on top of the mica with a high-viscosity optical adhesive (NOA63, Norland Products), followed by another round of UV exposure. Coverslips were separated to expose a freshly cleaved mica surface just before performing SB experiments.

### Formation of SBs

To prepare SB from M_2_R-containing liposomes onto the different substrates, we follow a published protocol with a few modifications (*33*). Coverslips (prepared as described in the previous sections) were placed inside a 37°C incubator in a pipette box containing wet tissue paper to keep the humidity constant. PLs were diluted to 0.1 mg/ml in M_2_R reconstitution buffer without TCEP before adding 75 μl of the sample onto the coverslips. After 1 min, an extra 150 μl of buffer was added. After another 2 min, the coverslips were washed by adding another 200 μl of buffer, followed by careful mixing, removing, and adding more buffer. This procedure was repeated three times.

### SB TIRF experiments

A separate chamber for TIRF experiments on microscopy coverslips was exchanged for the FBM chamber, keeping the rest of the microscope the same (fig. S3A). The TIRF chamber was designed to hold a Pellin-Broca prism with a 20-mm square aperture (ADB-20, Thorlabs) on top of which the samples (coverslips) were mounted using 10 μl of immersion oil (Type F immersion liquid, Leica Microsystems). After assembling the coverslip, a 3D-printed ring (TIRF-cup) was secured with vacuum grease and filled with IB for imaging (fig. S3A). Imaging was done as described before for FBM experiments, except that longer exposures were used (120 ms for mica and quartz and 130 ms for pegylated quartz).

### hTRAAK determination of *P*_o_

For *P*_o_ determination experiments, POPE:POPG (3:1 weight ratio) lipid bilayers were used to match the composition of hTRAAK liposomes. Liposomes were added on top of the bilayers and left to sink and fuse as described before, with the following differences: liposomes were diluted 50 times with IB followed by a twofold dilution with 1 M KCl to a final concentration of 0.1 mg/ml, and hTRAAK vesicles were allowed to sink for only 30 s before inducing fusion with KCl. This is because hTRAAK-containing liposomes have a stronger proclivity to fuse than all the other proteins we studied. Following the fusion of the PLs, the top chamber was perfused with 5 ml to remove unfused vesicles. Imaging using a scanned-focused laser was then performed as described above. Currents under the voltage clamp were measured during the whole procedure. To calculate the number of hTRAAK channels in the bilayer, videos were cropped to a region of the bilayer with homogeneous focus and illumination. Then, uTrack software was used to detect particles within the cropped videos. The number of detections was plotted as a function of time (frames) for each video to estimate the bleaching rate (fig. S8). Then, the average number of particles from different frames was calculated up to a frame that showed less than a 5% reduction in the number of particles. The number of detections was divided by the area of the (cropped) frames, which was calculated from the coordinates of the farthest detected particles, to obtain the density of detections. This density was then multiplied by the area of the bilayer, measured using the ROI area measurement feature on the NIS-elements software, and by a correction factor (LR) for the labeling efficiency (15%) to obtain the total number of channels (*N*) on the bilayer. Potassium currents were measured at 50 mV and divided by *N* and the single-channel current for hTRAAK at that voltage (3.6 pA) to obtain the *P*_o_ (*41*). hTRAAK tension titration experiments were done as described previously without any modification (*39*). We note that an important assumption in the determination of *P*_o_ is that incorporated TRAAK channels are functional.

### Tension activation studies of hTRAAK

FB pressurization and tension estimation were done as described previously (*39*). Briefly, hTRAAK-containing POPE:POPG (3:1 weight ratio) lipid bilayers were pressurized using a hydrostatic manometer connected to the bottom side of the experimental chamber (fig. S1). During pressure pulses, both hTRAAK currents and the bilayer capacitance were simultaneously measured every 100 ms (10 Hz). Bilayer capacitances were then converted to bilayer areas using the specific capacitance of the bilayer (μF/cm^2^), measured at the beginning of each experiment from an image of the unpressurized bilayer and its respective capacitance. Radii of curvature (*R_c_*) were then calculated from the areas (*A*) using the equation $R_{c}=\frac{A}{\sqrt{4\;A\;\pi -4\;a^{2}\pi^{2}}}$, where a is the radius of the hole on the plastic film where the bilayer was formed. Bilayer tensions (γ) were calculated from the radii of curvature and applied pressures (Δ*P*) using the Young-Laplace equation, $\Delta P=2\;\gamma \left(\frac{1}{R_{c}}\right)$.

### Phase-separation experiments

DphPC (4.4 mg; Avanti Polar Lipids), 2 mg of DSPC (Avanti Polar Lipids), and 1 mg of cholesterol (Co; Avanti Polar Lipids) in chloroform were mixed to a final 2:1:1 molar ratio. At this point, 250 ng of DSPE-AF488 (ammonium salt) was added to indicate the ordered phase. The lipid mixture was dried under argon gas and left under vacuum for at least 2 hours before resuspending into a mixture of decane:butanol (9:1) to a final concentration of 22 mg/ml. PBs were formed as described above. After imaging with two channels, hTRAAK trajectories were obtained using uTrack software and were overlayed on the first frame of the lipid channel using MATLAB (MathWorks). Experiments were done in IB at room temperature.
