# Supplementary material for: Freestanding bilayer microscope for single-molecule imaging of membrane proteins
Source: Sci Adv. 2024 Jun 21;10(25):eado4722. doi: 10.1126/sciadv.ado4722 (PMC11192074; doi:10.1126/sciadv.ado4722)
Supplement: Supplementary file 1 — Figs. S1 to S8 Legends for movies S1 to S8 [file sciadv.ado4722_sm.pdf]

Science Advances
AAAS

Supplementary Materials for

Freestanding bilayer microscope for single-molecule imaging of membrane proteins

Gonzalo Pérez-Mitta *et al.*

Corresponding author: Roderick MacKinnon, mackinn@rockefeller.edu

Sci. Adv. **10**, eado4722 (2024)
DOI: 10.1126/sciadv.ado4722

The PDF file includes:

Figs. S1 to S8
Legends for movies S1 to S8

Other Supplementary Material for this manuscript includes the following:

Movies S1 to S8

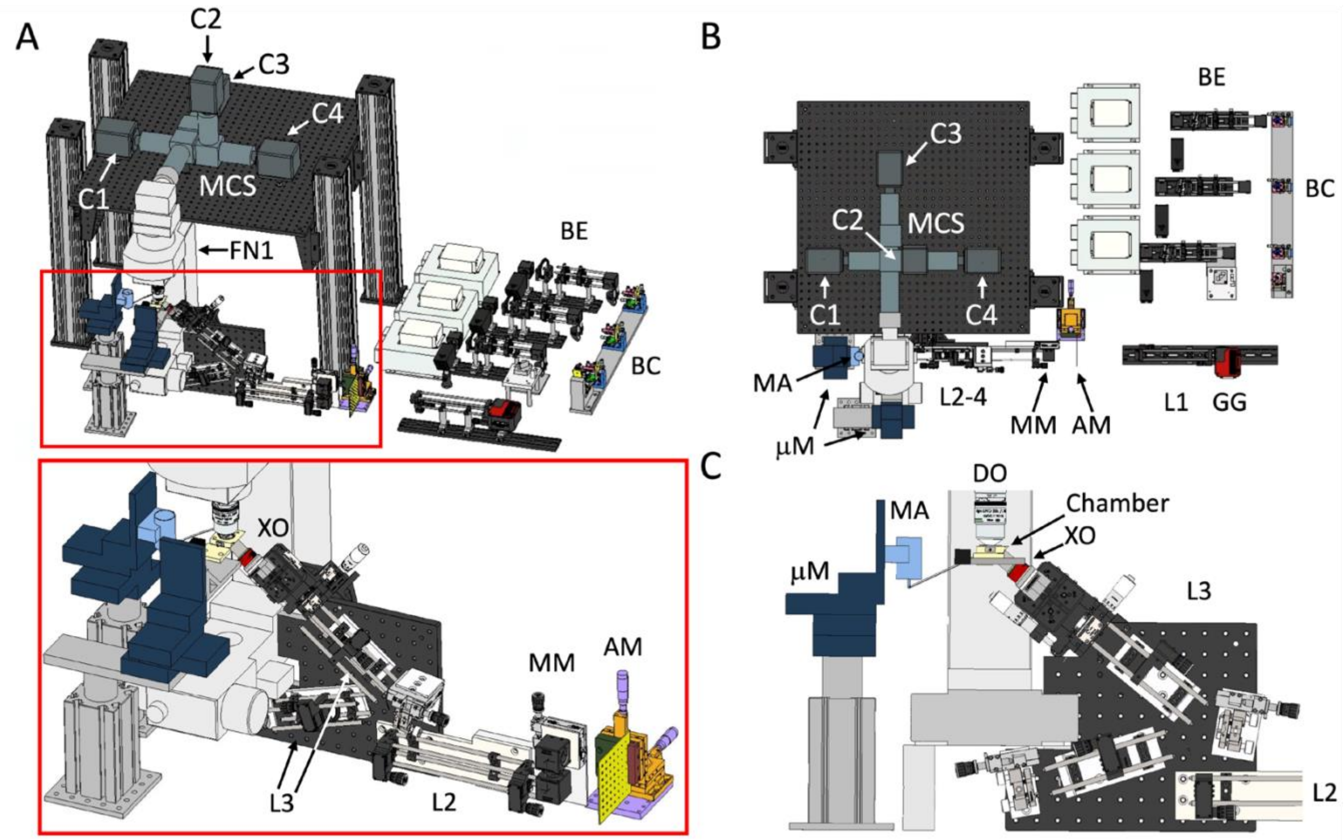

Figure S1. Views of the optomechanical model of the FBM. A. Top-front view. A magnified view of the excitation optics and the experimental chambers is shown in the red box. **B.** Top view. **C.** Cross-sectional view of the excitation optics and experimental chamber. Abbreviations stand for BE: Beam

expanders, BC: Beam (laser) combiner, GG: Galvo-Galvo scanner, L1-L3: Optical relays, AM: Apodization mask, MM: movable mirror, mM: micromanipulator, MA: manometer, XO: excitation objective, DO: detection objective, FN1: Upright microscope from Nikon (model FN1), MCS: multi-camera splitter, C1 to C4: cameras.

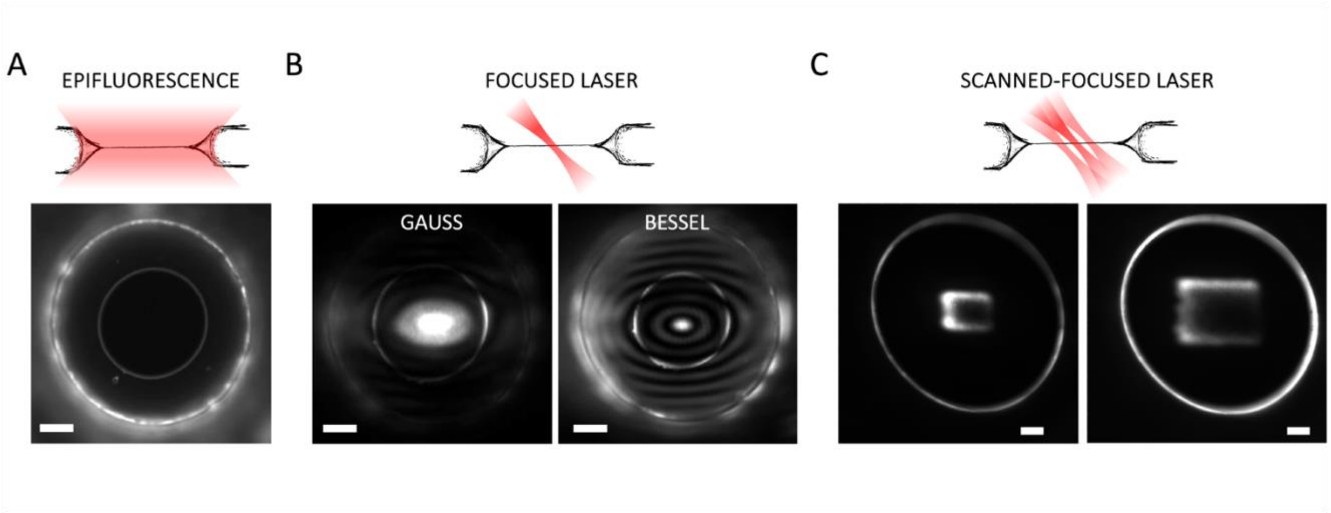

Figure S2. Comparison of different modes of illumination of a POPE: POPG (3:1 by weight) bilayer with Rhodamine-DOPE (0.01% w/w). A. Epifluorescence illumination. **B.** Focused-laser illumination with a Gauss (left) and Bessel (right) beam. **C.** Scanned focused-laser illumination using a scanning amplitude of 50 (left) and 100 (right) mV. Images A and B were taken on the same bilayer, and C was on a different one. Scale bars: 10 μm.

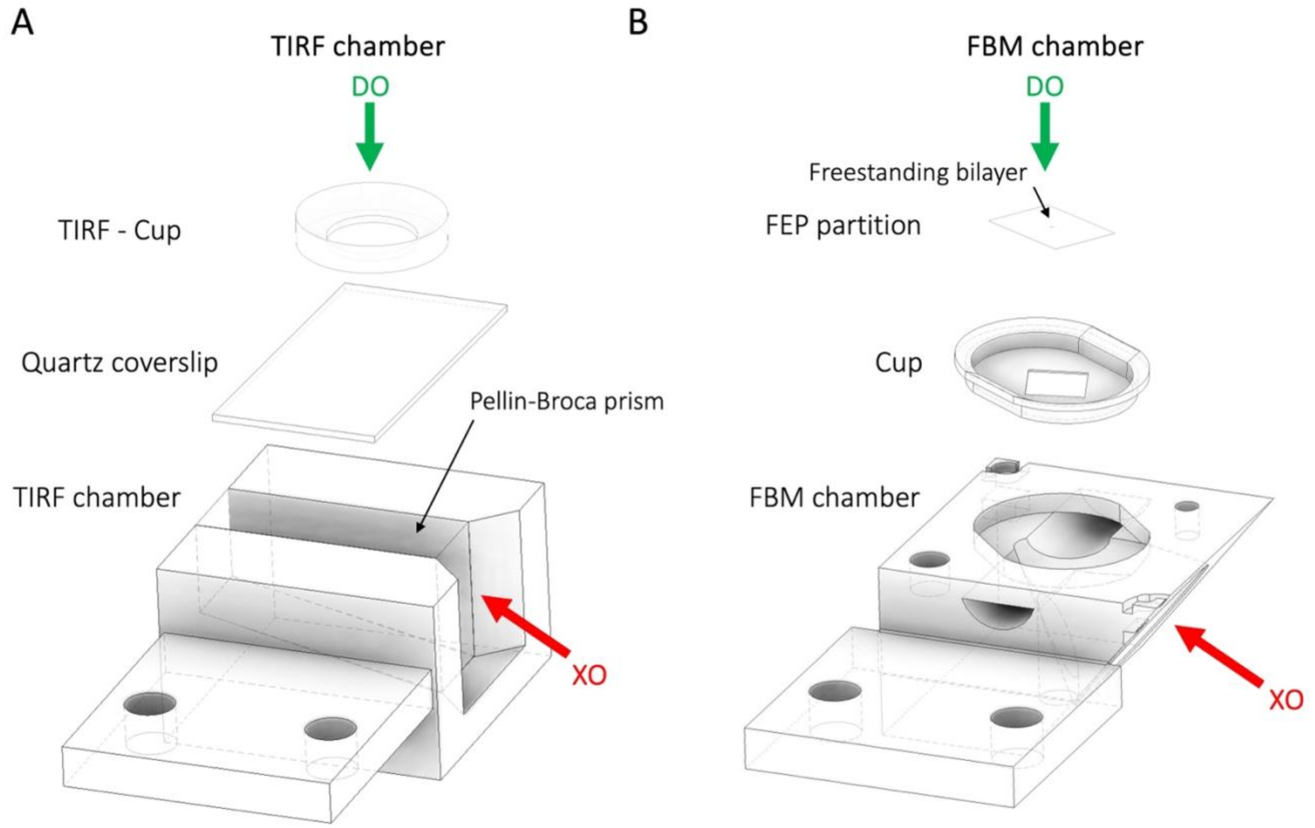

Figure S3. Optomechanical models of FBM and TIRF chambers. A. TIRF chamber. Amplified view of the TIRF chamber used for the SB experiments depicting the quartz coverslip on which SBs were formed and the TIRF cup that contains the imaging buffer. The coverslip is coupled to a Pellin-Broca prism through immersion oil. Red and green arrows are used to represent the directions of the incoming excitation (XO) and detection optics (DO), respectively. **B. FBM chamber.** Amplified views of the FBM chamber depicting the exchangeable cup that separates the top and bottom chamber and the FEP partition where the freestanding bilayer is formed.

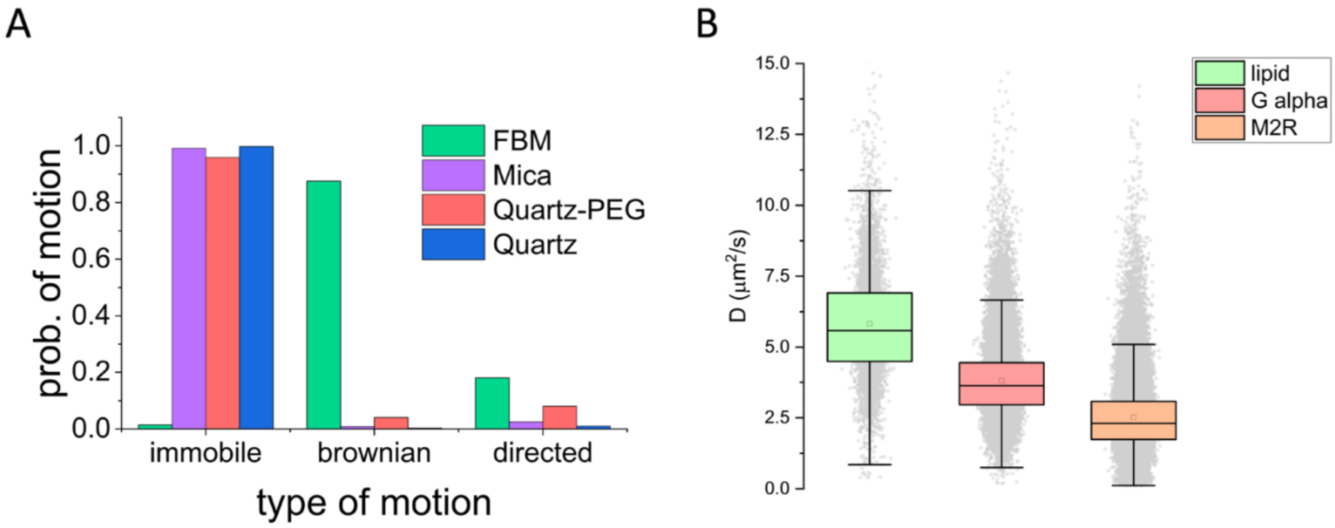

Figure S4. Single-particle-tracking (SPT) experiments. A. Classification of the data on M_2R shown in figure 2B. Results show a significant difference between the dominant type of motion in the FBM (Brownian) and SBs (immobile). **B. Diffusion coefficients (D) for DOPE-Cy5 (green), $G_{\alpha i}$ (red) and M_2R (orange) in the FBM**. The mean, 25;75 percentile (Box) and 5;95 percentile (bars) are shown overlaying the data. $G_{\alpha i}$ and M_2R were labeled with LD655 through Sfp site-directed labeling and with NbALFA-LD655, respectively.

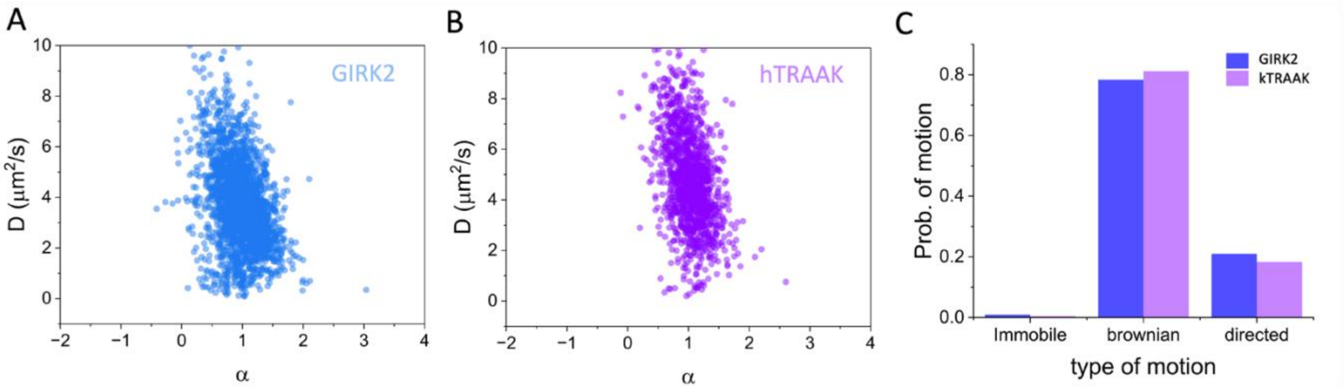

Figure S5. Single-particle-tracking (SPT) experiments. A. Diffusion coefficients (D) vs. anomalous coefficient (α) for GIRK2 (A) and hTRAAK (B). C. Classification of the tracks shown in panels A and B for GIRK2 (blue) and hTRAAK (violet). The results indicate that both proteins remain mobile

in FBM experiments. GIRK2 and hTRAAK where labeled with NbALFA-LD655 and LD655-NHS, respectively.

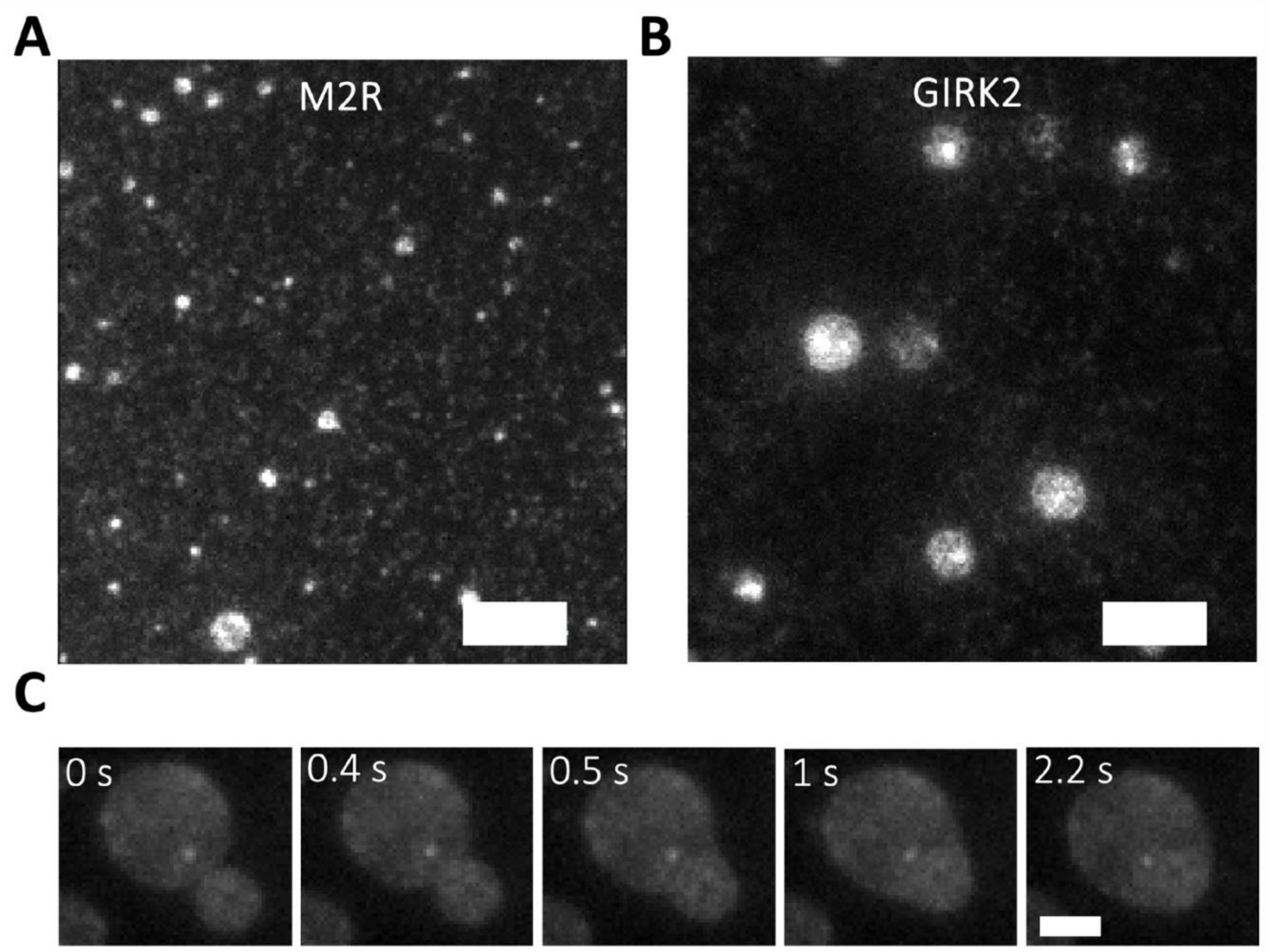

Figure S6. Planar protein aggregates are observed in FBs after proteoliposome fusion. Aggregates of 1-10 μm are routinely observed after vesicle fusion for **M_2R (A)** and **GIRK2 (B). C. A time sequence showing the fusion of two planar aggregates of GIRK2.** Scale bars: 10 μm (A, B) 4 μm (C).

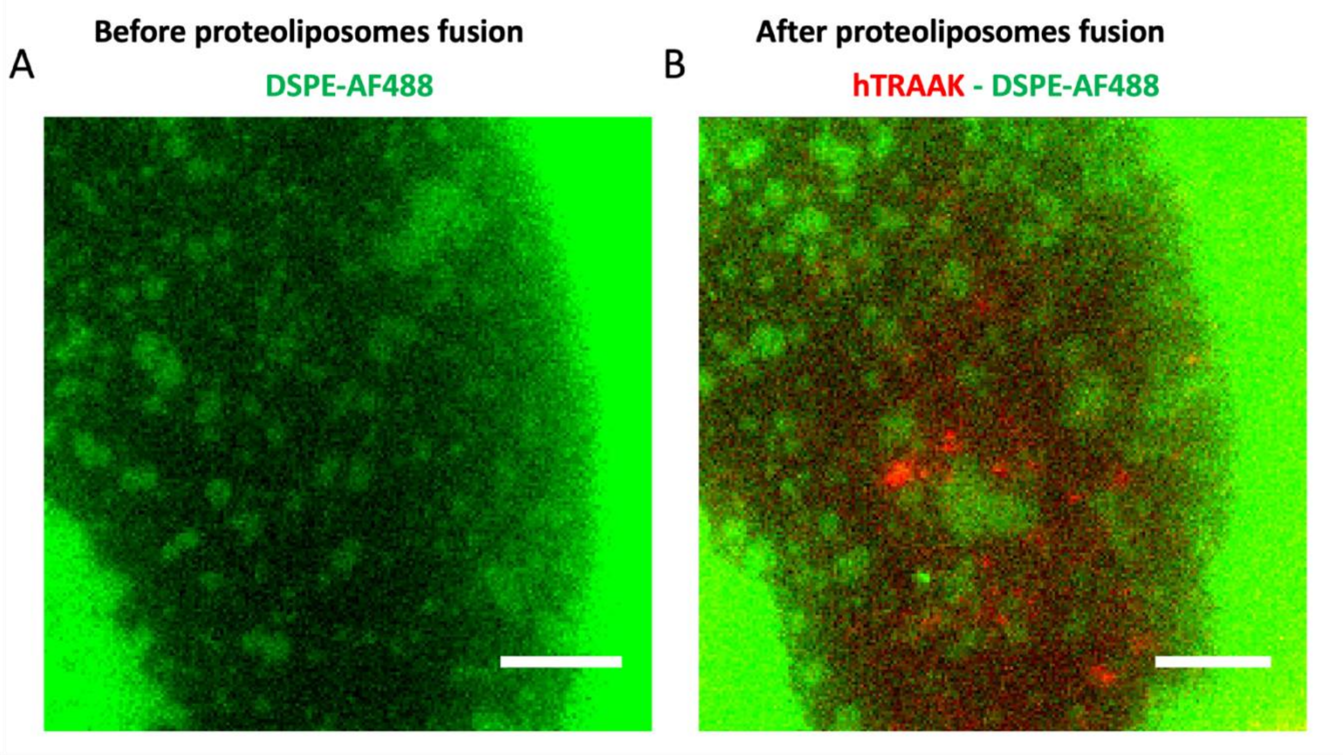

Figure S7. Representative image of a DphPC: DSPC: cholesterol (2: 1 :1 molar ratio) bilayer before (A) and after (B) the fusion of proteoliposomes containing hTRAAK (red). DSPE-AF488 (green) partitions into the ordered phase. Scale bars: 10 μm.

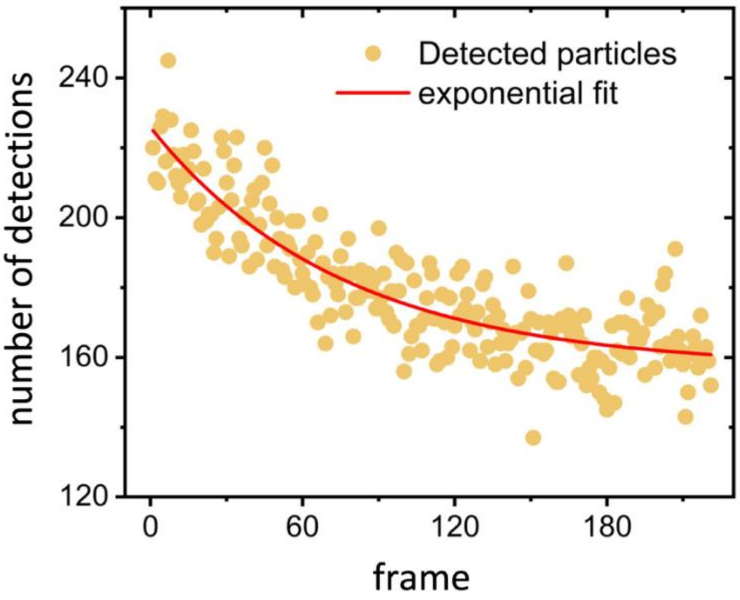

Figure S8. Number of hTRAAK particles detected per frame during a FBM video. Each frame lasts 30 ms. An exponential fit to the data is shown as a red line (r^2=0.99).

Videos were converted and compressed from 12 bit (.nd2 format) to 8 bit (.mp4 format) to ease their visualization with common video players.

video 1. Freestanding bilayer containing LD655-labeled hTRAAK observed under epifluorescence. Frame rate: 50 Hz.

video 2. Freestanding bilayer containing LD655-labeled hTRAAK observed under focused-laser illumination. Frame rate: 50 Hz.

video 3. Quartz-supported bilayer observed under TIRF illumination. Frame rate: 8 Hz.

video 4. Mica-supported bilayer observed under TIRF illumination. Frame rate: 8 Hz.

video 5. Pegylated-Quartz-supported bilayer observed under TIRF illumination. Frame rate: 8 Hz.

video 6. Freestanding bilayer containing the G-protein coupled receptor M_2R labeled with NbALFA-LD655. Frame rate: 67 Hz.

video 7. Freestanding bilayer containing the G-protein alpha $G_{\alpha i1}$ labeled with LD655 through Sfp-mediated site-directed labeling. Frame rate: 68 Hz.

video 8. Freestanding bilayer containing the fluorescent lipid DOPE-Cy5. Frame rate: 50 Hz.
